# Dyspeptic symptoms and delayed gastric emptying of solids in patients with inactive Crohn’s disease

**DOI:** 10.1186/1471-230X-12-175

**Published:** 2012-12-07

**Authors:** Ana Carolina Mello Nóbrega, Bruno Roberto Silva Ferreira, Graciela Josué Oliveira, Kamila Maria Oliveira Sales, Armênio Aguiar Santos, Miguel Ângelo Nobre e Souza, Lúcia Libanês Bessa Campelo Braga, Luiz Ernesto de Almeida Troncon, Marcellus Henrique Loiola Ponte Souza

**Affiliations:** 1 Institute of Biomedicine of Brazilian Semi-Arid (INCT-IBISAB), Department of Medicine, School of Medicine, Federal University of Ceará, Rua Cel. Nunes de Melo, 1315, CEP: 60430-270, Fortaleza, CE, Brazil; 2 Institute of Biomedicine of Brazilian Semi-Arid (INCT-IBISAB), Department of Physiology and Pharmacology, School of Medicine, Federal University of Ceará, Rua Cel. Nunes de Melo, 1315, CEP: 60430-270, Fortaleza, CE, Brazil; 3 Department of Medicine, Ribeirão Preto Faculty of Medicine, University of São Paulo, Ribeirão Preto, 14049-900, São Paulo, Brazil; 4 Centro de Biomedicina, Faculdade de Medicina, Universidade Federal do Ceará, Rua Cel. Nunes de Melo, 1315, Rodolfo Teófilo, Fortaleza-CE, Brazil

**Keywords:** Dyspeptic, Gastric emptying, Crohn

## Abstract

**Background:**

Patients with Crohn’s disease (CD) have been shown to present dyspeptic symptoms more frequently than the general population. Some of these symptoms could be related to motility disorders to some degree. Then, we propose to investigate whether gastric emptying of solids in patients with inactive CD is delayed and to determine the relationships between gastric emptying and dyspeptic symptoms in inactive CD.

**Methods:**

Twenty-six patients with inactive Crohn’s disease, as defined by a Crohn’s Disease Activity Index (CDAI) < 150, underwent a gastric emptying test by breath test using ^13^C octanoic acid coupled to a solid meal and answered a validated questionnaire (The Porto Alegre Dyspeptic Symptoms Questionnaire) to assess dyspeptic symptoms. Patients with scores ≥ 6 were considered to have dyspepsia. The control group was composed by 19 age- and sex-matched healthy volunteers.

**Results:**

Patients with CD had a significantly longer t 1/2 and t lag (p<0.05) than the controls. CD patients with dyspepsia had significantly (p<0.05) prolonged gastric emptying when compared to patients without dyspeptic symptoms. When the individual symptom patterns were analyzed, only vomiting was significantly associated with delayed gastric emptying (p<0.05). There was no difference between the subgroups of patients with respect to gender, CDAI scores, disease location, clinical behavior (obstructive/obstructive) or previous gastrointestinal surgery.

**Conclusion:**

Delayed gastric emptying in inactive Crohn’s disease patients seems to be associated with dyspeptic symptoms, particularly vomiting, even without any evidence of gastrointestinal obstruction.

## Background

Crohn’s disease is a chronic condition of unknown etiology in which inflammatory process may involve any location of gastrointestinal tract [1]. Patients with Crohn’s disease have been shown to present dyspeptic symptoms more frequently than the general population [2]. Although not primarily a motility disorder, some of the symptoms of Crohn’s disease could be explained by the presence of inflammatory mucosal changes, tissue swelling and occasional obstruction, which may have important effects on gastrointestinal motility [3]. Disordered motility patterns, including delayed gastric emptying, visceral hypersensitivity and upper intestinal hypomotility, have been observed in patients and animals with inflammatory bowel diseases [4-8].

Recently, Kristinsson et al. described a series of four patients with Crohn’s disease who presented severe symptoms and signs of gastroparesis. They suggest that clinicians should consider impaired gastric emptying when evaluating patients with Crohn’s disease who present with symptoms of upper gut dysmotility [9]. However, the association between gastric emptying delay and the presence of mild dyspeptic symptoms in Crohn’s disease patients remains unclear.

Therefore, we decided to investigate whether gastric emptying of solids in patients with inactive Crohn’s disease is delayed and to determine the relationships between gastric emptying and dyspeptic symptoms in this condition.

## Methods

### Study subjects

The study population consisted of 26 patients (12 men and 14 women, mean age of 43 years old, ranging 26–67 years old) diagnosed with Crohn’s disease based on accepted radiological, endoscopic and histological criteria [1] that were followed up in the outpatient clinics of the Walter Cantídio University Hospital, Fortaleza, State of Ceará, Brazil. Ten patients had ileocolitis, 9 patients had ileitis and in 7 patients, the disease was limited to the colon. In addition to the disease location, patients were also evaluated with respect to age, gender, weigh, height, disease behavior (obstructive or nonobstructive), age at presentation of Crohn’s disease [10,11], status of Helicobacter pylori infection, current treatment (steroids, 5-ASA derivatives, azathioprine/6-MP and anti-TNF agents) and previous abdominal surgery. All patients had no evidence of inflammatory activity, with Crohn’s Disease Activity Index [12] < 150 (mean: 47; range: 6–104) at the time of the study, with no use of corticosteroids in the last month. Nineteen healthy volunteers (7 men and 12 women; mean age 43 years old; range 19–73 years old) comprised a control group. All subjects signed consent forms to take part in the study, which was approved by the local Ethical Committee (protocol number: 049.07.08; date: 10/14/2008).

Subjects were instructed to avoid using any medications known to affect digestive motility (like proton pump inhibitors, domperidone, metoclopramide) or a diet with ^13^C enrichment (e.g., corn flour and pineapples) [13] in the week before the study. Subjects presenting with diabetes mellitus, thyroid disorders, autoimmune disorders, renal failure, chronic obstructive lung disease, egg protein allergy, gastric ulcer and gastric adenocarcinoma were excluded from the study after the appropriate investigations.

### Symptom questionnaire

Before the gastric emptying test, patients and healthy subjects were requested to complete a previously reported and validated questionnaire named The Porto Alegre Dyspeptic Symptoms Questionnaire (PADYQ) [14], which is an instrument of quantitative analysis of dyspeptic symptoms. This questionnaire is a unidimensional instrument that has been shown to have high levels of internal consistency, reproducibility, responsiveness, face validity, discriminant validity, and concurrent validity. This 11-question instrument assesses the frequency (score 0–4), duration (score 0–3), and intensity (0–5) of five dyspeptic symptoms (pain in upper abdomen, nausea, vomiting, upper abdominal bloating and early satiety) during the preceding 30 days. The score ranges from 0 (no symptoms) to 44 (severe symptoms). Patients with a total score of 6 or higher were considered to have dyspepsia [14].

### Gastric emptying studies

A gastric emptying test for solids was performed using a previously validated ^13^C octanoic acid breath test (^13^C-OABT) [15-17]. After an overnight fast, each patient received a 250 Kcal meal consisting of 60 g of white bread, 5 g of margarine and 1 egg (the yolk of which was labeled with 100 mg of ^13^C octanoic acid and sodium salt). The meal was ingested in 10 minutes and was followed immediately by 150 mL of water. Breath samples were obtained from the subjects exhaling into closed aluminized plastic bags before the meal administration (baseline measurement) and then at 15-minute intervals for 4 hours. During the test, patients were advised to remain seated and refrain from physical activity. Both the equipment (IRIS II - ^13^C-Breath Test System) and substrate (^13^C octanoic acid) used were provided by Wagner Analysen Technik GmbH, Bremen, Germany. The t lag (minutes) was defined the time with maximum speed of gastric emptying after ingestion of the test meal, the t 1/2 (minutes) was defined the time when first half of the ^13^C-labelled substrate dose of the test meal has been metabolized. Delayed gastric emptying were defined as a t 1/2 above 200 minutes, and t lag above 150 minutes, taking into account the manufacture’s reference (http://www.wagner-bremen.de), as well as data from previous studies [15].

### Helicobacter pylori assessment

Each patient was assessed for the presence of H. pylori infection. Patients without dyspeptic symptoms were submitted to a standard ^13^C urea breath test. Patients with dyspeptic symptoms underwent an upper gastrointestinal endoscopy with biopsy specimens collected from the gastric antrum and corpus for a rapid urea test and histological analysis.

### Data analysis

To analyze the relationship between gastric emptying and dyspeptic symptoms in Crohn’s disease patients, we subdivided the patients according to their total score in the PADYQ into two subgroups: dyspeptic (score ≥ 6) and non-dyspeptic (score < 6).

### Statistical analysis

The clinical characteristics of the patients (e.g., gender, presence of colitis and H. pylori infection) were compared by Fisher’s exact test. Statistical analysis of clinical and gastric emptying data was conducted by means of nonparametric and parametric tests using the Mann–Whitney U test and Student’s t-test, respectively, as needed. In Crohn’s disease dyspeptic patients, the individual scores of dyspeptic symptoms (PADYQ) were correlated with the presence of a delay in gastric emptying (t 1/2 > 200 minutes) by using Fisher’s exact test. Pearson rank correlation coefficients were used to assess the relationship between motility data and PADYQ scores. For all analysis, P < 0.05 was considered to be statistically significant. Statistical analysis was performed using a commercial software program (GraphPad Prism version 3.0; San Diego, CA, USA).

## Results

### Clinical characteristics of the study population

In general, the characteristics of the Crohn’s disease patients and healthy individuals were similar. The patient group had a mean age 43 years old (range 26–67 years old) and the control group had a mean age of 43 years old (range 19–73 years old), with no significant difference (p=0.98). Also, there were no differences between these two groups with respect to weight (patients = 63 Kg and controls = 68,5 Kg; p=0.19) and gender: among the patients, 14/26 (53.8%) were women, which was not significantly different (p=0,53) from the proportion found in the control group, 12/19 (63.2%).

Table 1 shows the differences between the two subgroups of patients with Crohn’s disease. In the group without dyspeptic symptoms, there were more patients (81.8%) that used immunosuppressant drugs (azathioprine/6-MP) for at least 3 months than the group of Crohn’s disease with dyspepsia (40%, p=0.04). There were not statistical differences between the two subgroups regarding age, weight, gender, age at presentation of disease, presence of colitis, disease behavior (obstructive/non obstructive), previous abdominal surgery, H. pylori infection, use of 5-ASA derivatives or CDAI scores. In the both group of Crohn’s patients, they did not take corticoids in the last month.

**Table 1:** Demographic and clinical characteristics of subgroups of Crohn’s disease patients with and without dyspepsia

	**Crohn’s disease without dyspepsia (n=11)**	**Crohn’s disease with dyspepsia (n=15)**	**P**
Age in years (mean ± s.e.m)	41.7 ± 3.5	44.1 ± 3.1	0.60
Weigh in Kg (mean ± s.e.m)	67.0 ± 4.2	60.5 ± 2.8	0.27
Gender (number of women and %)	5 (45.5%)	9 (60.0%)	0.36
Age at presentation (number < 40 years and %)	10 (91%)	9 (60.0%)	0.09
Patients with Colitis (number and %)	8 (72.7%)	9 (60.0%)	0.40
Patients with obstructive behavior (number and %)	8 (72.7%)	7 (46.7%)	0.17
Patients with previous abdominal surgery (number and %)	5 (45,5%)	5 (33.4%)	0.41
Patients with Helicobacter pylori (number and %)	6 (54.5%)	5 (33.4%)	0.22
5-ASA (number and %)	5 (45.5%)	11 (73.4%)	0.15
Azathioprine / 6-MP (number and %)	9 (81.8%)	6 (40.0%)	0.04*
CDAI scores (mean ± s.e.m)	36.6 ± 6.1	54.5 ± 9.7	0.25

### Gastric emptying test results

**Figure 1:**
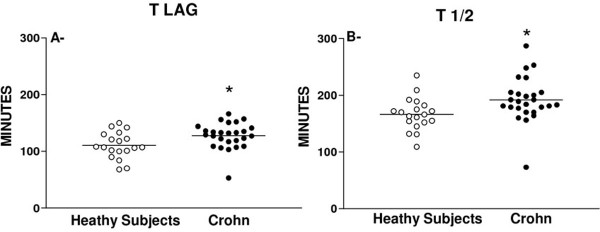
**Distribution of individual values for the t lag (panel A) and t 1/2 (panel B) in healthy subjects and patients with Crohn’s disease.** The horizontal bars represent mean values. (*) P<0.05 (Student’s t-test).

**Figure 2:**
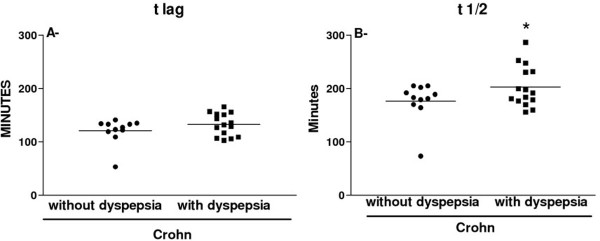
**Distribution of individual values for the t lag (panel A) and t 1/2 (panel B) in Crohn’s disease patients with or without dyspepsia.** The horizontal bars represent mean values. (*) P= 0.0446 (Student’s t-test).

Figure 1 presents the gastric emptying data of patients and healthy subjects and shows that Crohn’s disease patients had both t 1/2 (mean±SEM= 192.0 ± 7.7 minutes) and t lag (mean±SEM= 127.7 ± 4.5 minutes) values significantly longer (p<0.05) than those found in healthy controls (t 1/2 mean±SEM= 166.5 ± 6.6 minutes; t lag mean±SEM= 110.7 ± 5.3 minutes). When only the data from the Crohn’s disease patients were analyzed (Figure 2), we found (see panel B) that the symptomatic patients (PADYQ ≥ 6) had a significantly (p<0.05) prolonged gastric emptying (t 1/2 mean±SEM= = 203.2 ± 9.9 minutes) when compared with patients without symptoms (t 1/2 mean±SEM= = 176.6 ± 11.1 minutes). However, there was no statistical difference in the t lag (panel A) between the two subgroups with (mean±SEM= 132.8 ± 5.4 minutes) or without dyspeptic symptoms (mean±SEM= 120.8 ± 7.3 minutes).

### Relationships between dyspeptic symptoms and gastric emptying parameters

**Figure 3:**
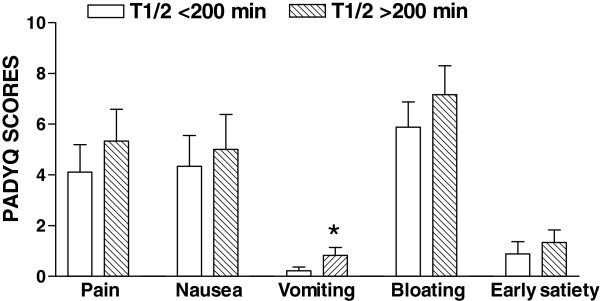
**Relationship between symptom severity and gastric emptying in patients with Crohn’s disease and dyspeptic symptoms.** The figure shows the PADYQ scores in the subgroups of patients with normal (t 1/2 < 200 min) or delayed gastric emptying (t 1/2 > 200 min). (*) P<0.05 (Student’s t-test).

When the scores of dyspeptic symptoms (PADYQ) were analyzed in relation to the presence of delayed gastric emptying (t 1/2 > 200 minutes) in dyspeptic Crohn’s disease patients, we found that only vomiting had a significant association with the presence of retarded gastric emptying. Moreover, a significant positive linear correlation was found only between the values for t 1/2 and the PADYQ scores for vomiting (p=0.048). However, there were no significant differences in the pain, nausea, bloating and early satiety scores between the subgroups of patients with and without delayed gastric emptying (Figure 3).

There was not significant correlation between the PADYQ scores (pain, nausea, vomiting, bloating and early satiety) and the presence of t lag delayed (> 150 minutes) (data not shown).

## Discussion

Patients with Crohn’s disease have more dyspeptic symptoms than the general population, even when there is no evidence of inflammatory activity [2,9,18,19]. Disorders in gastrointestinal motility have been observed in patients and in experimental models of Crohn’s diseases [4-8]. However, the association between gastric dysmotility and the presence of dyspeptic symptoms in these patients had not been previously investigated. Our study demonstrated that there was a delay in gastric emptying in inactive Crohn’s disease patients when compared with healthy individuals, and this gastric motility disorder seems to be associated with the presence of dyspeptic symptoms. In addition, we found a significant association between delayed gastric emptying and vomiting, even without any evidence of gastrointestinal obstruction.

Our results show that patients with inactive Crohn’s disease had significantly prolonged gastric emptying half time and lag time measured by ^13^C-OABT compared with healthy controls, which is in accordance with results published elsewhere [6,8]. Annese et al. demonstrated that gastric emptying, measured by a scintigraphic study, was slowed in patients with nonobstructive Crohn’s disease [6]. In addition, Keller et al. demonstrated that patients with Crohn’s disease had delayed gastric emptying, measured by a standardized ^13^C-octanoic acid breath test, and suggested that this disorder might partly be caused by excessive cholecystokinin (CCK) release [8]. The delayed gastric emptying in inactive Crohn’s disease could be due to a decrease in the antroduodenal contraction rate, as described by Annese et al. in 1997, which showed that in the fed state, the inactive Crohn’s disease patients had antrum hypomotility characterized by a decrease in the number of contractions [4]. Another possible explanation for these findings could be an increase in gastric compliance in these patients. However, at present and to the best of our knowledge, there are no studies available concerning gastric compliance in patients with Crohn’s diseases. The role of the intestinal inflammatory process in upper motility was evaluated by Schepper et al. who showed delayed gastric emptying in experimental models of acute colitis [7], which could be another explanation for the presence of gastric motility disorders away from the inflammatory site. However, despite the fact that our patients had no clinical evidence of intestinal inflammatory activity, as shown by very low CDAI scores, we cannot rule out the possibility of the presence of a mild level of inflammation in these patients.

Our results showed that Crohn’s diseases patients with dyspeptic symptoms had the t 1/2 time more prolonged than the patients without dyspeptic symptoms. However, there was no statistical difference in the t lag between the two subgroups. Then, we can infer that Crohn’s disease patients do not have major disturbances in gastric accommodation. The association between dyspeptic symptoms and delayed gastric emptying has been studied in other conditions, such as functional dyspepsia, and it has been found that only 23-26% of functional dyspeptic patients had delayed gastric emptying [20,21]. We herein observed delayed gastric emptying in 40% of the patients with Crohn’s disease and dyspeptic symptoms. There may be other pathophysiological mechanisms involved in the genesis of dyspeptic symptoms in Crohn’s disease such as visceral hypersensitivity, impaired accommodation and disturbed antral-duodenum contractility [22]. In accordance, Faure and Giguère already showed that in children with Crohn’s disease in remission suffering from chronic functional abdominal pain, the rectal sensory threshold for pain, measured by barostat, was significantly lower compared to normal subjects [5]; if this is true for visceral hypersensitivity, this might be a possible explanation for the presence of dyspeptic symptoms in our patients.

We found that Crohn’s disease patients without dyspepsia used more often immunosuppressant drugs (azathioprine-6MP) than patients with dyspepsia (p=0.04). This finding could indicate a more complete mucosal healing in these patients, which could not be expressed by a difference in CDAI scores between these two subgroups.

We observed that the particular symptom of vomiting was significantly associated with delayed solid emptying (Figure 3). In regards to dyspeptic symptoms, is important to note that our findings are in accordance with the observations by Sarnelli et al. who showed an association of delayed solid meal emptying rate with vomiting in patients with functional dyspepsia [20]. Despite the major differences between the studies, the presence of similar associations between the vomiting symptom and delayed gastric emptying confirms the strength of the possible link between pathophysiological mechanism and symptom complex; in the case of Crohn’s disease patients, duodenal obstruction could be a possible explanation for this association. Duodenal obstruction is not a rare condition, yet none of our patients displayed radiological or endoscopic evidence of it. Also, delayed gastric emptying has been previously demonstrated in patients with nonobstructive Crohn’s disease [6]. Besides, there were no differences between the two subgroups of patients with respect to the presence of obstruction or previous abdominal surgery.

Upper endoscopy performed in dyspeptic Crohn’s disease patients presented normal or minor endoscopic findings such as enanthematous gastritis. No significant differences in H. pylori infection were detected in Crohn’s disease patients with or without dyspeptic symptoms. The precise mechanisms of dyspeptic symptoms in patients with inactive Crohn’s disease are still unknown, but our findings suggest that impaired gastric emptying, clinically traduced as gastroparesis, could explain at least part of these symptoms.

## Conclusion

In summary, our results indicate that prolonged gastric emptying in inactive Crohn’s disease patients seems to be associated with dyspeptic symptoms. The closest association was found between delayed gastric emptying and vomiting, even without any evidence of gastrointestinal obstruction. This finding may be of clinical relevance to symptomatic patients, who could possibly provide benefit with the use of immunosuppressant drugs (azathioprine/6-MP). However, more studies need to be performed, including a larger sample of patients, in order to confirm our hypothesis.

## Competing interests

The authors declare that they have no competing interests.

## Authors’ contributions

ACMN: participated in the conception, performed the data collection, and write the manuscript. BRSP, GJO and KMOS participated in implementation of the study, data collection. AAS and MANS participated in the statistical analysis, interpretation and critical writing of the manuscript. LEAT and LLCB: performed critical writing. MHLPS: participated in conception, design, implementation, coordination of the study and critical writing. All authors have read and approved the final manuscript.

## Pre-publication history

The pre-publication history for this paper can be accessed here:

http://www.biomedcentral.com/1471-230X/12/175/prepub
